# Peripheral and central compensatory mechanisms for impaired vagus nerve function during peripheral immune activation

**DOI:** 10.1186/s12974-019-1544-y

**Published:** 2019-07-19

**Authors:** Anna Kobrzycka, Paweł Napora, Brandon L. Pearson, Krystyna Pierzchała-Koziec, Rafał Szewczyk, Marek Wieczorek

**Affiliations:** 1 Department of Neurobiology, Faculty of Biology and Environmental Protection, University of Lodz, Lodz, Poland; 2 Department of Environmental Health Sciences, Mailman School of Public Health, Columbia University, New York, USA; 3 Department of Animal Physiology and Endocrinology, University of Agriculture, Krakow, Poland; 4 Department of Industrial Microbiology and Biotechnology, Faculty of Biology and Environmental Protection, University of Lodz, Lodz, Poland

**Keywords:** Vagus nerve, Vagotomy, Prostaglandin E2, PGE2, Compensatory mechanisms, CNS communication, LPS, Elevated plus maze test, Sickness behavior

## Abstract

**Background:**

Determining the etiology and possible treatment strategies for numerous diseases requires a comprehensive understanding of compensatory mechanisms in physiological systems. The vagus nerve acts as a key interface between the brain and the peripheral internal organs. We set out to identify mechanisms compensating for a lack of neuronal communication between the immune and the central nervous system (CNS) during infection.

**Methods:**

We assessed biochemical and central neurotransmitter changes resulting from subdiaphragmatic vagotomy and whether they are modulated by intraperitoneal infection. We performed a series of subdiaphragmatic vagotomy or sham operations on male Wistar rats. Next, after full, 30-day recovery period, they were randomly assigned to receive an injection of *Escherichia coli* lipopolysaccharide or saline. Two hours later, animal were euthanized and we measured the plasma concentration of prostaglandin E2 (with HPLC-MS), interleukin-6 (ELISA), and corticosterone (RIA). We also had measured the concentration of monoaminergic neurotransmitters and their metabolites in the amygdala, brainstem, hippocampus, hypothalamus, motor cortex, periaqueductal gray, and prefrontal medial cortex using RP-HPLC-ED. A subset of the animals was evaluated in the elevated plus maze test immediately before euthanization.

**Results:**

The lack of immunosensory signaling of the vagus nerve stimulated increased activity of discrete inflammatory marker signals, which we confirmed by quantifying biochemical changes in blood plasma. Behavioral results, although preliminary, support the observed biochemical alterations. Many of the neurotransmitter changes observed after vagotomy indicated that the vagus nerve influences the activity of many brain areas involved in control of immune response and sickness behavior. Our studies show that these changes are largely eliminated during experimental infection.

**Conclusions:**

Our results suggest that in vagotomized animals with blocked CNS, communication may transmit via a pathway independent of the vagus nerve to permit restoration of CNS activity for peripheral inflammation control.

## Background

The vagus nerve is a prominent interoceptive pathway due to its complicated anatomical structure and extensive projection range [1, 2]. It is commonly accepted that in addition to its active participation in regulating the immune system, the vagus nerve is also part of the key pathway transmitting information about immune system activation to the CNS [3–8]. However, it is currently not known whether its role is indispensable. Ghia et al. (2007) showed that vagus nerve have a protective effects in colitis; however, they indicate that about 2 weeks after vagotomy, some compensatory changes in inflammatory response occur [9]. Such observations are supported by fact that the neural route of communication between immune and CNS systems, via sensory endings of the peripheral nerves, is just one of a few ways that mediators of inflammatory response (e.g., cytokines, prostaglandins) influence the CNS. In contrast, the humoral route involves the impact of proinflammatory mediators on capillary endothelial cells, which are constituents of the blood-brain barrier (BBB). These cells produce secondary mediators (e.g., PGE2), which act directly on the receptors on neurons or trigger the activation of microglia. In addition, inflammatory mediators have the potential to cross to the brain within circumventricular organs where BBB is leaky [4, 5, 10, 11] and subsequently acts on astrocytes and microglia to release PGE2 [12–14].

Acute and chronic neuropathies of the vagus nerve branches are known in certain clinical conditions such as viral upper respiratory infections [15], syphilis of the nervous system [16], during periods of alcohol abuse [17], or in type 2 diabetes [18]. Edwards et al. (2008) suggest even that the most common and the earliest manifestation of diabetic neuropathy involves the vagus nerve [19]. Given the role that this cranial nerve plays in the immune-CNS communication, it can be hypothesized that organisms develop compensatory mechanisms to permit interoceptive functions in states of vagus pathology. Given the prominent functions of cyclooxygenases (COXs) activity in immune-CNS relations [7, 20, 21], we hypothesized that they can “take over” functions of the impaired vagal pathway, exacerbating, among others, peripheral prostaglandin synthesis.

Sickness behavior and other concepts regarding the mutual influence of the nervous system on the immune one and vice versa, prompted researchers to study the communication and exchange of information between them [22–24]. Research on the neural pathway, in particular the vagus nerve, determined that peripheral activation of the immune system, reaching the CNS via the vagus nerve, initiates a number of metabolic changes, including prominent neurotransmission alterations [25–29] and leads, among others, to behavioral changes [30–35]. A number of research is focused at variable called vagal tone. The physiological level of vagal tone is crucial for allostasis of cardiac and digestive systems. It can be measured using heart rate variability (HRV)—the direct effect of vagal tone. It was discovered that vagal tone is also a predictor of emotional and behavioral responsiveness. Porges at al. (1994) generalized that “higher vagal tone and proper suppression of vagal tone during and attention-demanding task are related to better performance” and it is crucial for getting focused during tasks [36]. Porges also described the decreased vagal tone as a stress predictor [37]. In neuro-immune communication context, Bonaz et al. (2016) summarize that low vagal tone has a proinflammatory effect—increased plasmatic cortisol, TNF-α, and epinephrine levels [38, 39].

The physiological anti-inflammatory effect of vagal activity is associated with noradrenergic connection between vagal sensory nuclei and hypothalamus. Immunosensory signals from vagus afferents lead to the hypothalamic-pituitary-adrenal axis (HPA axis) activation and glucocorticoid secretion, indirectly modulating the activity of the activated immune system [40]. The same signal stimulates the central autonomic network (CAN) and subsequently both, sympathetic and parasympathetic part of the autonomic nervous system. Activation of the first one results in norepinephrine secretion; in tissue affected by inflammation and in lymphoid organs, regulating the immune cells activity [41]. Stimulation of the parasympathetic part of the CAN directly influence the activity of immune cells in organs exposed to pathogens (e.g., larynx, trachea, bronchi, stomach, small and large intestine, liver) through the anti-inflammatory cholinergic pathway, which is a part of the inflammatory reflex [42–44].

To date, the mechanisms that are able to compensate for impaired vagus nerve function are equivocal. This missing knowledge is particularly important, given emerging evidence of altered vagal function in the pathogenesis and potential therapy in a range of diseases such as obesity [45], Parkinson’s disease [46], epilepsy [47], asthma [48], gastrointestinal pathologies [49, 50], and inflammation in the respiratory [51] or circulatory system [52]. However, this central emphasis on the vagus nerve requires a delicate and detailed understanding of mechanisms associated with vagus nerve stimulation (VNS) and obstruction through mechanical or chemical vagotomy (VGX).

In order to identify compensatory mechanisms for experimental impaired immunosensory functions of the vagus nerve, we performed a series of experiments to determine how subdiaphragmatic vagotomy and peripheral immune activation act on rats behavior, neurotransmitters metabolism within brain structures associated with sickness behavior, and finally peripheral markers of the inflammatory response and the HPA axis activity. We hypothesized that a compensatory mechanism would prevent observable differences between vagotomized and sham-operated rats after immune activation in a manner suggesting that parallel pathways are sufficient to link peripheral immune activity and brain physiology. Finally, we expected that this compensation following vagotomy would increase the activity/efficiency of the enzymatic route of contact between these two systems.

## Methods

Three-month-old male Wistar rats (Medical University of Białystok, Poland), with an initial weight of 250 g ± 10 g (*N* = 123), were individually housed in breeding cages, with free access to water and feed (Purina granules) under artificial lighting conditions (a 12-h day-night cycle, light on at 7.00 AM). The room temperature was set to 21–22 °C and humidity to 60–65%. The animals were habituated for 7 days to the conditions in the animal facility.

### Surgical procedure and experimental groups

A surgical plane of general anesthesia was obtained with an intraperitoneal injection of Innovar plus (6 μl/g body weight) and local anesthesia with 2% lidocaine solution (0.5 ml/animal). A bilateral subdiaphragmatic vagotomy (VG, *n* = 54) was then performed as follows. After placing the animal on the back, an incision of 5 cm long in the abdominal integuments was made. The liver was gently displaced to expose the esophagus. Using the surgical magnifier, both branches of the vagus nerve (gastrointestinal and hepatic) were identified. A small fragment of the nerve was removed just below to the diaphragm (about 0.7 mm in length). Next, the abdominal muscles were sutured with surgical threads. The skin was also sutured and an antibacterial agent (Alu Spray, V.M.D.) was applied to the site to prevent post-operative infection and to facilitate wound healing. Sham-operated animals (SH, *n* = 54) were subjected to the same procedure; however, in this case, the vagus nerve branches were not cut. Following surgery, the animals were placed individually in cages, and after a 30-day recovery period were used in the experiment. Vagotomy surgeries were verified using retrograde tracing technique with Fluoro-gold, according to Powley et al. (1987) [25, 53]. The experiment also included a naive control group (CNT, *n* = 15), without any injections or surgical procedures.

Within all experimental groups, except for CNT, a single intraperitoneal injections of saline (100 μl i.p., 0.9% NaCl) or LPS (*Escherichia coli* 026:B6, 10 μg in 100 μl 0.9% NaCl, 100 μl i.p.) were administered.

### The experiments

Forty-eight animals (SH NaCl = 12, SH LPS = 12, VG NaCl = 12, and VG LPS = 12) were tested in elevated plus maze test (EPM), 120 min after the intraperitoneal injection of saline or LPS (a duration sufficient to produce molecular markers of inflammation, [28]). Animals were placed on the central zone of the arena, and following a 10-s delay for a recording was made for 5 min. Immediately after the EPM test, animals were euthanized in a special room, dedicated to euthanasia and tissue sampling. All behavioral tests and euthanization of the animals was carried out between 10:00 and 12:00 AM to avoid the impact of circadian corticosterone fluctuations on the test results.

For the second experiment, 75 rats were used. Sixty animals (SH NaCl = 15, SH LPS = 15, VG NaCl = 15, and VG LPS = 15) were euthanized 125 min after the intraperitoneal injections. In parallel, from naive control group (CNT = 15), animals were euthanized. Immediately after decapitation, blood and the following brain structures were collected from each animal: amygdala (AM), brainstem (BS), hippocampus (HI), hypothalamus (HPT), motor cortex (CTX), medial prefrontal cortex (PFM), and periaqueductal gray (PAG). Each of the collected brain structures was weighed and homogenized using an ultrasonic homogenizer (BioBlock Scientific) for 15 s in 150 μl (200 μl for brainstem) homogenization solution (0.4 mM Na_2_S_2_O_5_, 0.6 mM HClO_4_). The homogenate was centrifuged at 12,000 rpm, for 15 min, at 4 °C. After centrifugation, at least 100 μl of the supernatant was collected from each sample, transferred to chromatographic tubes, and frozen at − 72 °C until chromatographic analysis.

Plasma was obtained by centrifuging the trunk blood collected on EDTA (1 ml/100 μl of Na_2_EDTA); 4000 rpm for 10 min at 4 °C. Obtained plasma was frozen and stored at − 72 °C until future analysis.

### Plasma biochemical analysis

Plasma interleukin 6 (IL-6) was determined using the Human IL-6 ELISA Kit, from Invitrogen, according to the manufacturer’s instructions. The concentration of corticosterone in plasma of the tested animals was determined using radioimmunoassay (Corticosterone Rat/Mouse kit, DRG International), according to the manufacturer’s instructions. Plasma prostaglandin E2 (PGE2) concentration was determined using HPLC-MS method. An AB Sciex 4500 QTRAP mass spectrometer (AB Sciex, USA), coupled with an Eksigent microLC 200 System (Eksigent, USA) were employed for analysis. Chromatography separation was conducted on an Eksigent C18-EP (0.5 mm × 50 mm × 3 μm, 120 Å) column with 50 °C temperature. Injection was made in the standard full loop mode resulting in 5 μl volume on column injection. The eluent consisted of 0.1% formic acid in water (A) and 0.1% formic acid in acetonitrile (B). The gradient used had a constant flow of 50 μl/min with 0.5 min of preflush conditioning, followed by 0.2 min in which 98% of eluent A was used and then a linear decrease to 2% of eluent A in 1.2 min and hold until 2.0 min. Initial conditions were restored from 2.1 to 2.5 min. The optimized microESI ions source parameters were as follows: CUR: 30; IS: − 4500/+ 5000 V; Temp: 400 °C; GS1: 28; GS2: 30; and ihe: ON. The MS/MS detection was made in multiple reaction-monitoring (MRM) mode. The following MRM pairs were applied: 351.2–271.1, 351.2–315.0 (negative ionization) for PGE2 and 393.1–373.2, 393.1–355.1 (positive ionization) for betamethasone. The quantitation was performed according to standard curves with betamethasone used as internal standard. The linearity of the quantification curves within the working range of the method covered concentration 0.05–10 ng/ml with a regression coefficient of *r* = 0.9997 for PGE2.

### Monoamines and their metabolites analysis

The concentration of the main monoamines; noradrenaline (NE), serotonin (5HT), and dopamine (DA) and their metabolites; 3-methoxy-4-hydroxyphenylglycol (MHPG), 5-hydroxyindoleacetic acid (5HIAA), 3,4-dihydroxyphenylacetic acid (DOPAC), and homovanillic acid (HVA) was determined in collected brain samples with RP-HPLC-ED method. The Agilent 1100 chromatographic system with Waters Spherisorb ODS-1 RP C-18 chromatographic column (4.6 × 250 mm) preceded by a Zorbax SB-C18 pre-column (4.6 × 12.5 mm) was used. Column temperature was set at 35 °C and mobile phase flow at 1 ml/min. The carbon working electrode was set at + 0.65 V, relative to the Ag/AgCl reference electrode. The mobile phase consisted of a phosphate buffer (pH 3.4) containing: 0.15 M NaH_2_PO_4_ × H_2_O, 0.1 M Na_2_EDTA, 0.5 mM Na_2_OSA, 0.5 mM LiCl, and addition of methanol (10%). The chromatographic data were analyzed using CHEMSTATION, REVISION-B.03.02, Agilent software.

### Behavior analysis

The EPM test arena was constructed of black painted wood and consisted of four 50-cm-long and 20-cm-wide cross-like arms—two closed, surrounded by walls, 40 cm height, and two open (without walls). All arms were extended from a central square, 20 × 20 cm. The maze was elevated 50 cm above the floor surface. We decided to change original dimensions of EPM arena, because we want to observe an animal mobility in addition to anxiety-related behavior. Tests were performed in scattered red light. After each test, the arena was cleaned with 70% ethanol. Trials were recorded and analyzed using video tracking software EthoVision XT 11.5 by Noldus.

### Statistical analysis of the data

The experiment was constructed for multivariate analysis of variance (MANOVA test). However, part of the biochemical data did not meet the criteria for parametric tests, even after transformations (at first Box-Cox, next natural logarithm, decimal logarithm, square root). For such data, we decided to use non-parametric analysis of variance (Kruskal-Wallis test) with Bonferroni correction. To keep a similar analysis model for all data, the data with normal distribution (Shapiro-Wilk test) were analyzed with parametric one-way ANOVA with Duncan post-hoc test. The following two-tailed sub-hypothesis were analyzed and displayed on the figures:

1. CNT vs. SH + NaCl i.p.: Sham operation and vehicle injection (procedural activities) do not affect the value of the analyzed variable.
2. SH + NaCl i.p. vs. VG + NaCl i.p.: Subdiaphragmatic vagotomy does not affect the value of the analyzed variable.
3. SH + NaCl i.p. vs. SH + LPS i.p.: LPS injection does not affect the value of the analyzed variable under control conditions.
4. VG + NaCl i.p. vs. VG + LPS i.p.: LPS injection does not affect the value of the analyzed variable in vagotomized animals.
5. SH + LPS i.p. vs. VG + LPS i.p.: Response to LPS injection is similar in vagotomized and sham-operated animals.

For behavioral variables, data were additionally analyzed in terms of time of the EPM test using the same statistical tests. One of the behavioral variables includes qualitative data, so we used the χ^2^ test. Statistically significant *p* value for all tests was determined to be at *p* < 0.05. The biochemical results were shown as a median ± interquartile range ± min-max, while behavioral data were shown as mean ± SEM. All statistical analysis was performed using the STATISTICA, version 13.3 (TIBCO Software Inc. 2017). To increase the clarity of the text, only a portion of the results of the statistical analyses are in the text itself. The results of the entire statistical analyses can be found in the Tables 1, 2, 3, and 4.

**Table 1:** Results of statistical analysis of the neurotransmitters utilization indexes in examined brain areas

Brain area/ratio	MHPG/NE	5HIAA/5HT	HVA/DA	DOPAC/DA
PAG	*F*_(4,34)_ = 1.66, *p* = 0.181	*F*_(4,26)_ = 2.10, *p* = 0.110	***H***_**(*4*,*32*)**_ = ***18***.***96***, ***p*** < ***0***.***001***	*F*_(4,28)_ = 0.22, *p* = 0.925
HPT	*F*_(4,35)_ = 0.38, *p* = 0.818, BC	***F***_**(*4***,***26***)_ = ***4***.***64***, ***p*** = ***0***.***006***	***F***_(***4***,***26***)_ = ***40***.***93***, ***p*** < ***0***.***001***, ***BC***	***F***_(***4***,***31***)_ = ***6***.***04***, ***p*** = ***0***.***001***
AM	*H*_(4,37)_ = 11.22, *p* = 0.024	***F***_(***4***,***24***)_ = ***12***.***57***, ***p*** < ***0***.***001***	***F***_(***4*,*29***)_ = ***13***.***82****,* ***p*** < ***0****.****001***, ***BC***	***F***_(***4****,****24***)_ = ***3***.***09***, ***p*** = ***0***.***035***
PFM	*F*_(4,36)_ = 1.82, *p* = 0.146	*H*_(4,29)_ = 4.86, *p* = 0.302	***F***_(***4***,***24***)_ = ***29***.***34***, ***p*** < ***0***.***001***	*F*_(4,32)_ = 2.05, *p* = 0.111, BC
CTX	*F*_(4,36)_ = 1.30, *p* = 0.289, BC	***F***_(***4***,***29***)_ = ***5***.***70***, ***p*** = ***0***.***002***	***F***_(***4***,***32***)_ = ***13***.***94***, ***p*** < ***0***.***001***	*F*_(4,34)_ = 0.67, *p* = 0.619, √
HI	*F*_(4,35)_ = 0.75, *p* = 0.565, BC	*F*_(4,22)_ = 0.89, *p* = 0.485	***H***_(***4***,***32***)_ = ***11***.***93***, ***p*** = ***0***.***018***	*F*_(4,33)_ = 1.65, *p* = 0.185, √
BS	*H*_(4,38)_ = 9.77, *p* = 0.045	***H***_(***4***,***43***)_ = ***14***.***24***, ***p*** = ***0***.***007***	***H***_(***4****,****35***)_ = ***11***.***13***, ***p*** = ***0***.***025***	*F*_(4,29)_ = 0.89, *p* = 0.478

*F*—ANOVA test, *H*—Kruskal-Wallis test, BC—normal distribution after Box-Cox transformation, √—normal distribution after square root transformation, standard font—statistical insignificant, bolded and italicized font—statistical significant. *AM* amygdala, *BS* brainstem, *HI* hippocampus, *HPT* hypothalamus, *CTX* motor cortex, *PFM* medial prefrontal cortex, *PAG* periaqueductal gray

**Table 2:** Results of statistical analysis of the neurotransmitters concentrations in examined brain areas

Brain area/conc. [ng/g]	NE	DA	5HT
PAG	*F*_(4,39)_ = 1.89, *p* = 0.130	***H***_(***4***,***44***)_ = ***14***.***39***, ***p*** = ***0***.***006***	*F*_(4,8)_ = 0.23, *p* = 0.914, LN
HPT	*F*_(4,39)_ = 0.52, *p* = 0.719	*F*_(4,39)_ = 0.58, *p* = 0.677	*F*_(4,39)_ = 0.65, *p* = 0.632
AM	*F*_(4,39)_ = 1.68, *p* = 0.174	*F*_(4,38)_ = 1.92, *p* = 0.126	***F***_(***4***,***38***)_ = ***2***.***91***, ***p*** = ***0***.***034***, ***BC***
PFM	*F*_(4,37)_ = 0.47, *p* = 0.761	*F*_(4,37)_ = 0.97, *p* = 0.436	***H***_(***4***,***25***)_ = ***6***.***06***, ***p*** = ***0***.***001***, ***LN***
CTX	*F*_(4,38)_ = 4.17 *p* = 0.007, BC	***F***_(***4***,***38***)_ = ***5***.***21***, ***p*** = ***0***.***002***, ***BC***	***F***_(***4***,***38***)_ = ***3***.***60***, ***p*** = ***0***.***014***
HI	*F*_(4,39)_ = 0.82, *p* = 0.521	***F***_(***4***,***37***)_ = ***3***.***08***, ***p*** = ***0***.***028***, ***LN***	*F*_(4,38)_ = 0.71, *p* = 0.591
BS	***F***_(***4***,***39***)_ = ***4***.***90***, ***p*** = ***0***.***003***	***F***_(***4***,***38***)_ = ***64***.***46***, ***p*** < ***0***.***001***, ***BC***	*F*_(4,39)_ = 2.34, *p* = 0.072

*F*—ANOVA test, *H*—Kruskal-Wallis test, BC—normal distribution after Box-Cox transformation, LN—normal distribution after natural logar transformation, standard font—statistical insignificant, bolded and italicized font—statistical significant. *AM* amygdala, *BS* brainstem, *HI* hippocampus, *HPT* hypothalamus, *CTX* motor cortex, *PFM* medial prefrontal cortex, *PAG* periaqueductal gray

**Table 3:** Results of statistical analysis of behavioral variables in elevated plus maze test

Variable/time	1′	2′	3′	4′	5′
Distance moved (m)	*F*_(3,44)_ = 0.85, *p* = 0.475	*F*_(3,44)_ = 0.77, *p* = 0.516	*F*_(3,44)_ = 1.38, *p* = 0.261	***F***_(***3***,***44***)_ = ***4***.***66***, ***p*** = ***0***.***006***	***F***_(***3***,***44***)_ = ***3***.***31***, ***p*** = ***0***.***029***
Velocity (cm/s)	*F*_(3,44)_ = 0.92, *p* = 0.440	*F*_(3,44)_ = 0.75, *p* = 0.526	*F*_(3,44)_ = 1.38, *p* = 0.260	***F***_(***3***,***44***)_ = ***4***.***66***, ***p*** = ***0***.***006***	***F***_(***3***,***44***)_ = ***3***.***29***, ***p*** = ***0***.***029***
Activity (mean %)	*F*_(3,44)_ = 0.98, *p* = 0.412	*F*_(3,44)_ = 1.11, *p* = 0.354	*F*_(3,44)_ = 1.15, *p* = 0.339	***F***_(***3***,***44***)_ = ***2***.***73***, ***p*** = ***0***.***049***	***F***_(***3***,***44***)_ = ***2***.***82***, ***p*** = ***0***.***049***
Enter to closed (f)	***F***_(***3***,***44***)_ = ***3***.***86***, ***p*** = ***0***.***016***	*F*_(3,44)_ = 0.81, *p* = 0.493	*F*_(3,44)_ = 1.23, *p* = 0.309	*F*_(3,44)_ = 0.83, *p* = 0.479	*F*_(3,44)_ = 1.12, *p* = 0.351
Open/closed time ratio	*F*_(3,44)_ = 0.84, *p* = 0.482	*F*_(3,44)_ = 1.23, *p* = 0.312	*F*_(3,44)_ = 0.72, *p* = 0.547	***F***_(***3***,***44***)_ = ***2***.***91***, ***p*** = ***0***.***045***	*F*_(3,44)_ = 0.43, *p* = 0.731
Variable/group	SH NaCl	SH LPS	VG NaCl	VG LPS	
Distance moved (m)	***F***_(***4***,***55***)_ = ***6***.***04***, ***p*** < ***0***.***001***	*F*_(4,55)_ = 0.87, *p* = 0.491	*F*_(4,55)_ = 0.22, *p* = 0.926	*F*_(4,55)_ = 0.52, *p* = 0.714	
Velocity (cm/s)	***F***_(***4***,***55***)_ = ***5***.***99***, ***p*** < ***0***.***001***	*F*_(4,55)_ = 0.91, *p* = 0.466	*F*_(4,55)_ = 0.22, *p* = 0.928	*F*_(4,55)_ = 0,52, *p* = 0.724	
Activity (mean %)	***F***_(***4***,***55***)_ = ***5***.***84***, ***p*** < ***0***.***001***	*F*_(4,55)_ = 0.32, *p* = 0.864	*F*_(4,55)_ = 0.49, *p* = 0.737	*F*_(4,55)_ = 0.54, *p* = 0.708	
Enter to closed (f)	*F*_(4,55)_ = 1.94, *p* = 0.116	*F*_(4,55)_ = 1.89, *p* = 0.127	*F*_(4,55)_ = 0,71, *p* = 0.585	*F*_(4,55)_ = 1.52, p = 0.206	
Open/closed time ratio	*F*_(4,55)_ = 1.32, *p* = 0.273	*F*_(4,55)_ = 2.31, p = 0.072	*F*_(4,55)_ = 1.19, *p* = 0.324	*F*_(4,55)_ = 2.21, *p* = 0.078	

*F*—ANOVA test, standard font—statistical insignificant, bolded and italicized font—statistical significant. 1′,2′, 3′, 4′, 5′ —following minutes of the test, *SH* sham surgery, *VG* subdiaphragmatic vagotomy, *NaCl* single intraperitoneal vehicle injection, *LPS* single intraperitoneal LPS injection

**Table 4:** Results of statistical analysis of behavioral variables in endings of closed arms of the arena of EPM test, chosen after heat map observation

Variable/arm	Left		Right	
Entries to endings of closed arms (f)	*F*_(3,236)_ = 1.62, *p* = 0.186		***F***_(***3***,***236***)_ = ***2***.***91***, ***p*** = ***0***.***035***	
Time spending in endings of closed arms (s)	***F***_(***3***,***236***)_ = ***4***.***37***, ***p*** = ***0***.***005***		***F***_(***3***,***236***)_ = ***3***.***43***, ***p*** = ***0***.***018***	
Variable/group	SH NaCl	SH LPS	VG NaCl	VG LPS
Entries to endings of closed arms (f)	*F*_(1,128)_ = 2.82, *p* = 0.096	*F*_(1,88)_ = 0.32, *p* = 0.571	*F*_(1,128)_ = 2.28, *p* = 0.133	***F***_***(1,128)***_ ***= 10.55, p = 0.001***
Time spending in endings of closed arms (s)	*F*_(1,128)_ = 0.09, *p* = 0.766	***F***_(***1***,***88***)_ = ***4***.***76***, ***p*** = ***0***.***032***	***F***_(***1***,***128***)_ = ***10***.***63***, ***p*** = ***0***.***001***	***F***_(***1***,***128***)_ = ***8***.***18***, ***p*** = ***0***.***005***

*F*—ANOVA test, standard font—statistical insignificant, bolded and italicized font—statistical significant. *Left* ending of the left closed arm, *right* ending of the right closed arm, *SH* sham surgery, *VG* subdiaphragmatic vagotomy, *NaCl* single intraperitoneal vehicle injection, *LPS* single intraperitoneal LPS injection

## Results

### Biochemical changes in plasma (Fig. 1)

We observed specific changes in plasma IL-6 concentration (*F*_(4,54)_ = 27.14, *p* < 0.001). Administration of LPS caused a significant increase in IL-6 level, in both sham-operated (*p* < 0.001) and vagotomized animals (*p* = 0.010) confirming that the test animals developed inflammation within the peritoneal cavity. However, in vagotomized animals, this increase was significantly smaller (*p* < 0.001).

**Fig. 1:**
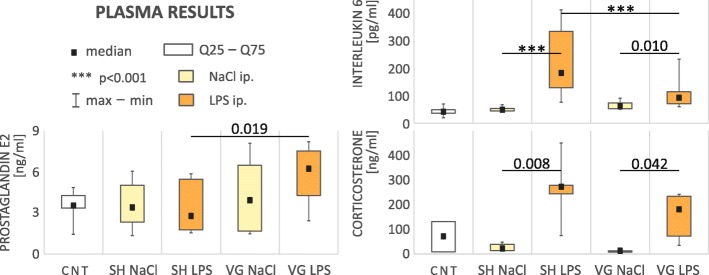
Results of analysis of selected plasma biochemical parameters. *CNT* naive control, *SH* sham surgery, *VG* subdiaphragmatic vagotomy, *NaCl* single intraperitoneal vehicle injection, *LPS* single intraperitoneal LPS injection. Vagotomized rats during early stages of inflammation have an elevated plasma PGE2 level and reduced level of IL-6. Vagotomy did not affect corticosterone level. Details in text

We observed statistically significant changes in plasma PGE2 concentration (*F*_(4,40)_ = 2.66, *p* = 0.046). PGE2 level in vagotomized rats after LPS administration was significantly higher compared to sham animals after the same treatment (*p* = 0.019).

A significant increase in plasma corticosterone concentration after LPS administration (*H*_(4,29)_ = 21.89, *p* < 0.001) was observed in both experimental groups; sham (*p* = 0.008) and vagotomized (*p* = 0.042). We did not observed a significant difference in plasma corticosterone level as a function of vagotomy within LPS-treated rats; however, it must be pointed out that adrenal reaction to LPS in vagotomized animals was smaller than in sham-operated rats.

### Activity of central neurotransmitters systems

#### Noradrenaline

**Fig. 2:**
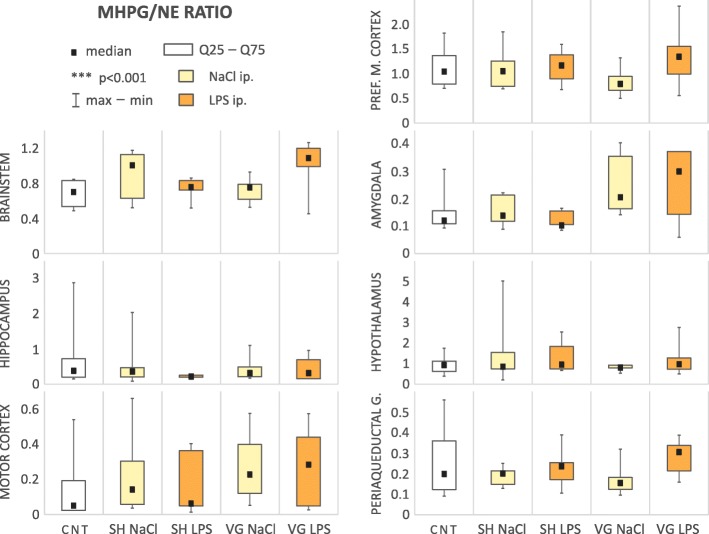
Noradrenaline utilization index. *CNT* naive control, *SH* sham surgery, *VG* subdiaphragmatic vagotomy, *NaCl* single intraperitoneal vehicle injection, *LPS* single intraperitoneal LPS injection. We did not observed any changes in MHPG/NE ratio

In the examined brain regions, there was no significant change of the noradrenaline utilization index (MHPG/NE ratio, Table 1, Fig. 2).

Differences in concentration of the noradrenaline (Table 2, Fig. 6a) were observed only in brainstem (*F*_(4,39)_ = 4.90, *p* = 0.003). LPS injection to sham-operated animals decreased (*p* = 0.049) noradrenaline level relative to vehicle; however, in vagotomized animals, this effect was not observed, probably because the neurotransmitter level was decreased after vagotomy itself (*p* = 0.028).

#### Serotonin

**Fig. 3:**
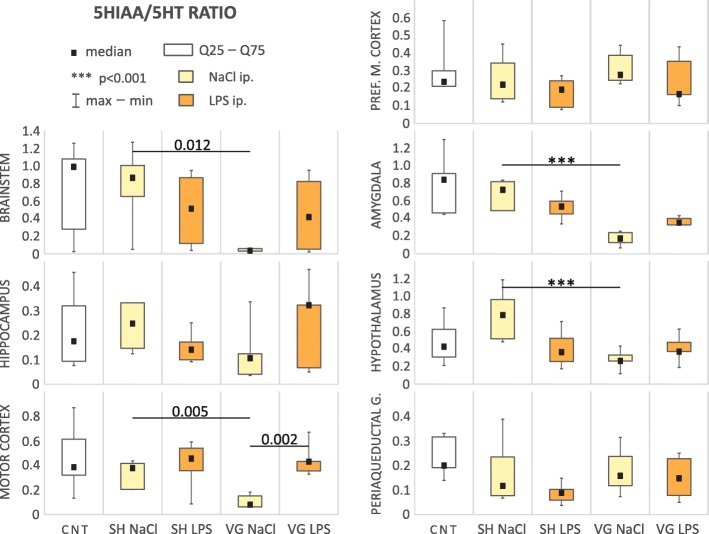
Serotonin utilization index. *CNT* naive control, *SH* sham surgery, *VG* subdiaphragmatic vagotomy, *NaCl* single intraperitoneal vehicle injection, *LPS* single intraperitoneal LPS injection. Vagotomy significant reduced 5HIAA/5HT ratio in brainstem, amygdala, hypothalamus, and motor cortex. During inflammation this effect seems to be neutralized, especially in motor cortex. Details in text

We observed a significant change in the serotonin utilization index (5HIAA/5HT ratio, Table 1, Fig. 3) within the brainstem (*H*_(4,43)_ = 14.24, *p* = 0.007), motor cortex (*F*_(4,29)_ = 5.69, *p* = 0.002), amygdala (*F*_(4,24)_ = 12.57, *p* < 0.001), and hypothalamus (*F*_(4,26)_ = 4.64, *p* = 0.006). In all of these areas, we observed that vagotomy caused a significant decrease of the 5HIAA/5HT ratio: brainstem (*p* = 0.012), motor cortex (*p* = 0.021), amygdala (*p* < 0.001), and hypothalamus (*p* < 0.001). Interestingly, experimental infection caused by intraperitoneal injection of LPS largely normalized the serotoninergic utilization index in these structures at a level similar (with no significant differences) to that observed in sham-operated animals. This tendency is particularly evident in motor cortex. In vagotomized rats in this area, the 5HIAA/5HT ratio was significantly higher than in vehicle-injected animals after LPS administration.

Changes in serotonin concentration (Table 2, Fig. 6b) were observed in the amygdala (*F*_(4,38)_ = 2.91, *p* = 0.034), motor cortex (*F*_(4,38)_ = 3.60, *p* = 0.014), and medial prefrontal cortex (*F*_(4,25)_ = 6.06, *p* = 0.001) also suggest altered serotoninergic neurotransmission after vagotomy. Serotonin concentration in medial prefrontal cortex of vagotomized animals was significantly lower than in sham ones both in vehicle (*p* = 0.001) and LPS (*p* = 0.009) injected groups. In motor cortex, vagotomy itself increased serotonin concentration (*p* = 0.001). However, after LPS injection, the level is significantly decreased (*p* = 0.026)—there was no statistical significant difference in serotonin concentration after LPS injection between sham and vagotomized animals. In the amygdala, we observe the cross-effect—LPS-injected vagotomized rats has significantly decreased serotonin level compared to vehicle-injected ones (*p* = 0.009) and to sham operated after LPS injection (*p* = 0.018).

#### Dopamine

**Fig. 4:**
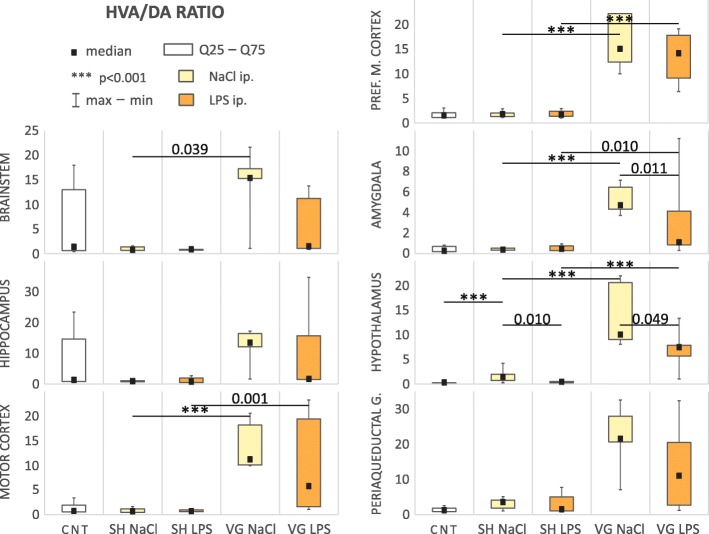
Dopamine to HVA utilization index. *CNT* naive control, *SH* sham surgery, *VG* subdiaphragmatic vagotomy, *NaCl* single intraperitoneal vehicle injection, *LPS* single intraperitoneal LPS injection. Vagotomy elevate HVA/DA ratio in all examined brain areas, in brainstem, motor cortex, prefrontal medial cortex, amygdala, and hypothalamus. Tendency to neutralized this changes is still observed, especially in amygdala and hypothalamus. Details in text

We observed changes in index utilization of dopamine relative to HVA (HVA/DA ratio, Table 1, Fig. 4) in the brainstem (*H*_(4,35)_ = 11.13, *p* = 0.025), motor cortex (*F*_(4,32)_ = 13.94, *p* < 0.001), medial prefrontal cortex (*F*_(4,24)_ = 29.34, *p* < 0.001), amygdala (*F*_(4,29)_ = 13.82, *p* < 0.001), and hypothalamus (*F*_(4,26)_ = 40.93, *p* < 0.001). Statistical test also indicate some significant difference between group in hippocampus (*H*_(4,32)_ = 11.93, *p* = 0.018) and periaqueductal gray (*H*_(4,32)_ = 18.96, *p* < 0.001); however, post-hoc test did not confirm this result in the first area, and in second reveal significant differences in comparisons from outside of our a priori comparisons.

Vagotomy strikingly and significantly increased the HVA/DA ratio in analyzed brain areas: brainstem (*p* = 0.039), motor cortex (*p* < 0.001), medial prefrontal cortex (*p* < 0.001), amygdala (*p* < 0.001), and hypothalamus (*p* < 0.001). After LPS injection, this index remained elevated in the motor cortex (*p* = 0.001), medial prefrontal cortex (*p* < 0.001), amygdala (*p* = 0.010), and hypothalamus (*p* < 0.001) of the vagotomized animals. This tendency was observed in other brain regions but failed to reach statistical significance. In the majority of regions, the ratio was still increased in vagotomized group also after LPS administration. However, we noted a tendency toward decreased HVA/DA ratio, which was increased as a result of vagotomy itself. This tendency was particularly evident in the amygdala (*p* = 0.011) and hypothalamus (*p* = 0.049). It also suggests that during infection, neurotransmitter activity in the CNS is putatively “normalized” in such a manner to efficiently regulate the intensity of peripheral inflammation, despite the vagotomy.

In hypothalamus—the first element of the HPA axis (or stress axis), we also observed a significant increase (*p* < 0.001) of the HVA/DA ratio in sham-operated and vehicle-injected animals, compared to naive control, and we connect this effect with increased contact with the experimenter, despite all rats having been handled prior to experimentation.

**Fig. 5:**
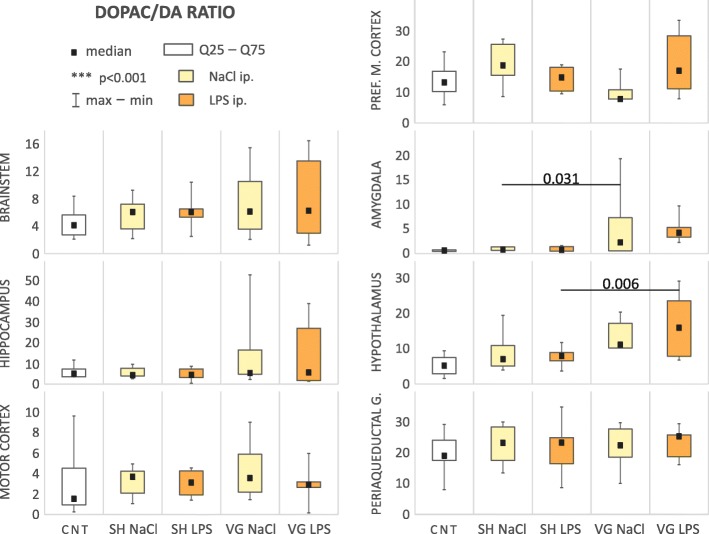
Dopamine to DOPAC utilization index. *CNT* naive control, *SH* sham surgery, *VG* subdiaphragmatic vagotomy, *NaCl* single intraperitoneal vehicle injection, *LPS* single intraperitoneal LPS injection. Vagotomy itself elevate DOPAC/DA ratio in amygdala. Again this effect is not observed during inflammation. However, we observed an elevated DOPAC/DA ratio in hypothalamus of vagotomized rats during inflammation. Details in text

Subdiaphragmatic vagotomy only modestly affected the utilization index of dopamine to DOPAC (DOPAC/DA ratio, Table 1, Fig. 5). Only within the amygdala (*F*_(4,24)_ = 3.09, *p* = 0.035) of vagotomized animals, DOPAC/DA ratio was significantly elevated, compared to sham-operated ones (*p* = 0.031). In turn in hypothalamus (*F*_(4,31)_ = 6.04, *p* = 0.001), this ratio was increased after LPS administration in vagotomized rats compared to sham-operated animals (*p* = 0.010), while vagotomy itself did not affect the ratio.

**Fig. 6:**
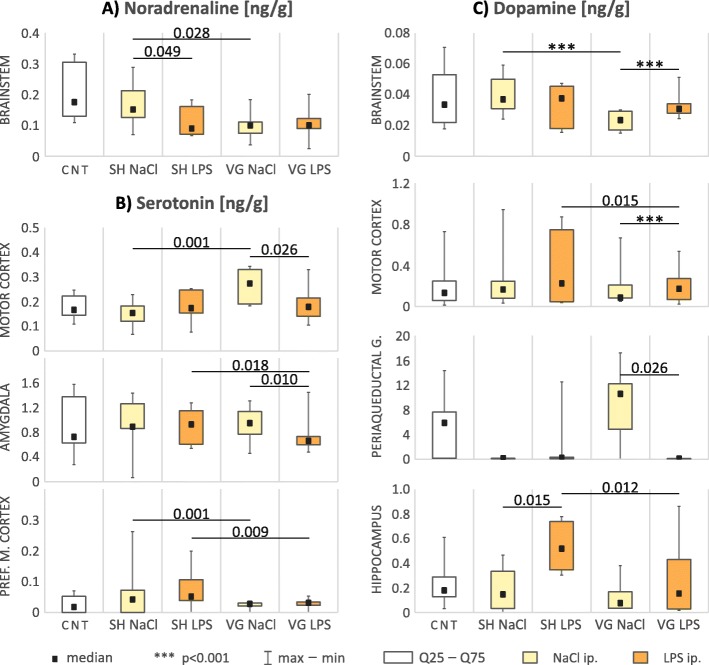
Concentration [ng/g of wet tissue] changes of monoaminergic neurotransmitters in examined areas. *CNT* naive control, *SH* sham surgery, *VG* subdiaphragmatic vagotomy, *NaCl* single intraperitoneal vehicle injection, *LPS* single intraperitoneal LPS injection. Figure shows results only for areas/neurotransmitter with statistical significant differences between groups. All of statistical data for whole tested variants are showed in Table 3. Details in text

Analysis of dopamine concentration (Table 2, Fig. 6c) also revealed changes between groups in the hippocampus (*F*_(4,37)_ = 3.08, *p* = 0.028), motor cortex (*F*_(4,38)_ = 5.21, *p* = 0.002), brainstem (*F*_(4,38)_ = 64.46, *p* < 0.001), and periaqueductal gray (*F*_(4,44)_ = 14.39, *p* = 0.006). In the motor cortex, vagotomy caused decreased dopamine concentrations (*p* = 0.002); however, after LPS injection, we can observe aforementioned tendency to “normalize” the dopamine level (*p* < 0.001). Dopamine concentration after LPS injection was significantly lower in hippocampus (*p* = 0.012) and brainstem *(p* < 0.001) of vagotomized animals, compare to sham ones. In the periaqueductal gray, the dopamine concentration was increased after vagotomy; however, after LPS injection, it lowered (*p* = 0.026) to a level similar to other groups.

### Behavioral activity (Table 3, Fig. 7)

#### Locomotor activity

Sham-operated and vehicle-injected rats displayed specific pattern of locomotor activity during EPM test. Statistically significant differences was observed in terms of distance moved (*F*_(4,55)_ = 6.04, *p* < 0.001), velocity (*F*_(4,55)_ = 5.99, *p* < 0.001), and activity (*F*_(4,55)_ = 5.84, *p* < 0.001). In the fourth minute, all of these parameter were significantly increased compare to the first 3 min of the test (4th vs. 1st distance moved *p* < 0.001, velocity *p* < 0.001; 4th vs. 2nd distance moved *p* < 0.001, velocity *p* < 0.001, activity *p* < 0.001; 4th vs. 3rd distance moved *p* = 0.015, velocity *p* = 0.016, activity *p* = 0.006). In the fifth minute, this tendency is still observed (5th vs. 1st distance moved *p* = 0.017, velocity *p* = 0.017, activity *p* = 0.001; 5th vs. 2nd distance moved *p* = 0.032, velocity *p* = 0.032, activity *p* = 0.020); however, there was no significant difference between this time point and the third minute.

**Fig. 7:**
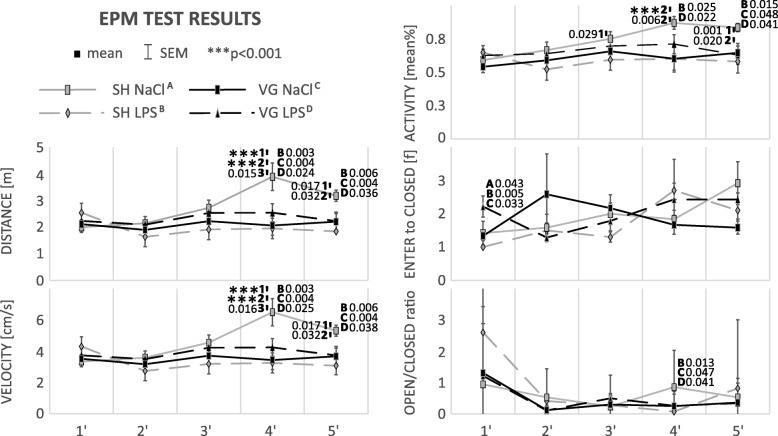
Locomotor activity (distance, velocity an activity), risk assessment (open/closed ratio), and anxiety (enter to closed) changes during elevated plus maze test. *SH* sham surgery, *VG* subdiaphragmatic vagotomy, *NaCl* single intraperitoneal vehicle injection, *LPS* single intraperitoneal LPS injection. During first 3 min of test, locomotor activity level was the same in all groups. However, in fourth and fifth minute, sham-operated and vehicle-injected rats (control) were more active. They were also more willing to take risky behaviors. LPS injection, subdiaphragmatic vagotomy, and combination of these two factors reduce this effect. Interestingly, only a combination of LPS injection and vagotomy causes a significant increase of anxiety level; however, this effect was observed only in first minute of the test. Details in text

Such model of the behavior was not observed in the rest of the groups, that is why these three parameters were also significantly different compared to other groups (distance moved 4th *F*_(3,44)_ = 4.66, *p* = 0.006, 5th *F*_(3,44)_ = 3.31, *p* = 0.029; velocity 4th *F*_(3,44)_ = 4.66, *p* = 0.006, 5th *F*_(3,44)_ = 3.29, *p* = 0.029; and activity 4th *F*_(3,44)_ = 2.73, *p* = 0.049, 5th *F*_(3,44)_ = 2.82, *p* = 0.049). Increases of these three parameters in sham, vehicle-injected group was attenuated in LPS-injected sham (distance moved 4th *p* = 0.003 and 5th *p* = 0.006, velocity 4th *p* = 0.003 and 5th p = 0.006, activity 4th *p* = 0.025 and 5th *p* = 0.015) and in subdiaphragmatic vagotomized groups (distance moved 4th *p* = 0.004 and 5th *p* = 0.004, velocity 4th *p* = 0.004 and 5th *p* = 0.004, activity 5th *p* = 0.048). LPS administration to the vagotomized group slightly weakened this effect; however, the tendency was still observed (distance moved 4th *p* = 0.024 and 5th *p* = 0.036, velocity 4th *p* = 0.025 and 5th *p* = 0.038, activity 4th *p* = 0.022 and 5th *p* = 0.041).

#### Risk assessment

Vagotomy or LPS-induced attenuation of locomotors activity is associated with risk assessment defined by ratio of time spending in open arms to closed ones. In the fourth minute of the test, open/closed ratio was higher in sham-operated and vehicle-injected animals (*F*_(3,44)_ = 3.86, *p* = 0.045) than in another groups (vs. SH LPS *p* = 0.013, VG NaCl *p* = 0.047, VG LPS *p* = 0.041).

#### Anxiety

Vagotomized and LPS-injected animals in the first minute of the test shown statistically significant increased level of anxiety trough more frequently entries to closed arms of the arena (*F*_(3,44)_ = 3.86, *p* = 0.016), compared to other groups (vs. SH NaCl *p* = 0.043, SH LPS *p* = 0.005, VG NaCl *p* = 0.033). In the following minutes, there were no statistically significant differences between groups in the anxiety level.

#### Heat map (Table 4, Fig. 8)

Observation of automatically generated heat maps by EthoVision Software revealed some potentially interesting preferences in animal behavior in the EPM test. According to this observation, we have defined additional zones—at the endings of the left and right closed arms, and we decided to look closer at the number of entries and time spent within it. Time spent in endings of the right (*F*_(3,236)_ = 3.43, *p* = 0.018) or left (*F*_(3,236)_ = 4.37, *p* = 0.005) closed arm was significantly different between groups. Vagotomized and vehicle-injected animals spent more time in ending of the right closed arm than sham-operated and vehicle-injected ones (*p* = 0.043). The same tendency is observed in sham-operated and LPS-injected group; however, result is insignificant (*p* = 0.068). Interestingly, vagotomized and LPS-injected animals, compared to these two groups, spent less time in the ending of the right closed arm (vs. SH LPS *p* = 0.009, VG NaCl *p* = 0.005) but more in the left one (vs. SH LPS *p* = 0.013, VG NaCl *p* = 0.010).

**Fig. 8:**
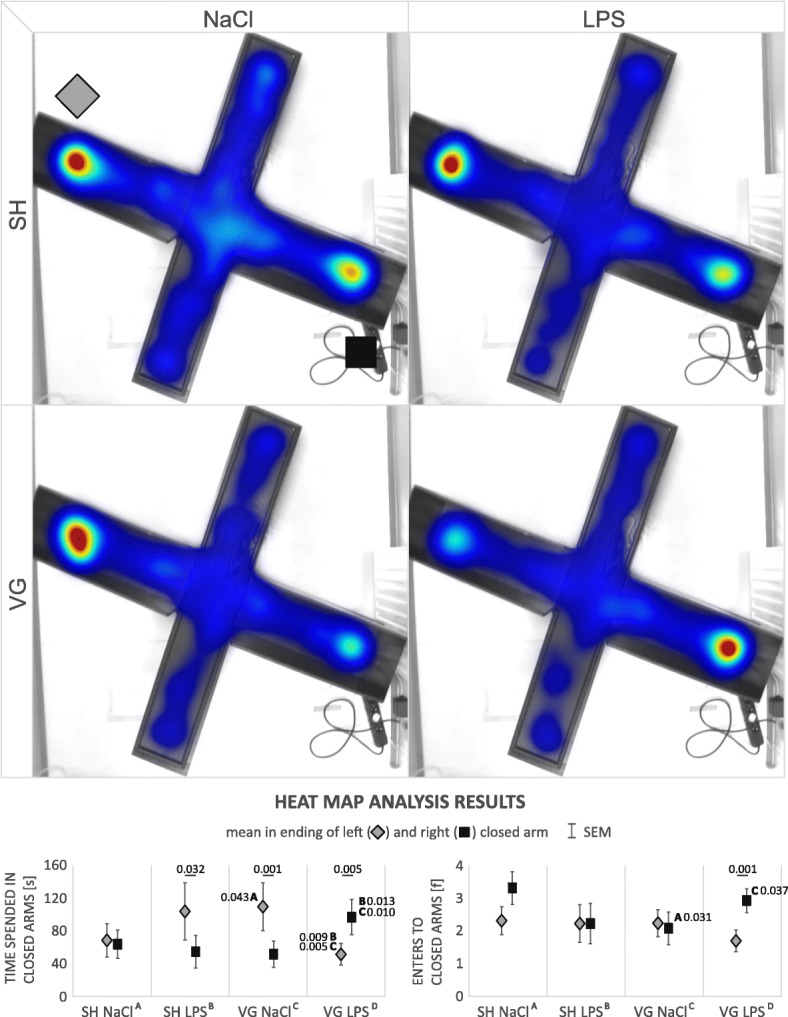
Heat map indicate some specific preference in rats behavior. *SH* sham surgery, *VG* subdiaphragmatic vagotomy, *NaCl* single intraperitoneal vehicle injection, *LPS* single intraperitoneal LPS injection. Statistical analysis showed that control rats (sham operated and vehicle injected) have no significant preference between endings of right or left closed arm, however animals after LPS injection or after vagotomy spend more time in ending of the left closed arm. Interestingly, vagotomized animals during inflammation more explore, and prefer to spend more time in the opposite ending of right closed arm. Details in text

Also, the number of entries to ending of the right closed arm was significantly different between groups (*F*_(3,236)_ = 2.91, *p* = 0.035). Vagotomized and vehicle-injected animals more frequently enters to the ending of the right closed arm than sham-operated and vehicle-injected ones (*p* = 0.031). Again, the same tendency is observed in the sham-operated and LPS-injected group; however, result is insignificant (*p* = 0.089). Vagotomized and LPS-injected animals enter to the ending of the right closed arm more frequently than vagotomized and vehicle-injected group (*p* = 0.037). On the other hand, we did not observe any differences in number of entries to ending of the left closed arm.

There was no significant difference in exploration of both areas (time spending or number of entries to ending of the left or right closed arm) in sham-operated and vehicle-injected group. LPS-administrated sham animals still explored (entered) both arms endings in the same way, but they spent more time in the left one (*F*_(1,88)_ = 4.76, *p* = 0.032). The same tendency was displayed in vagotomized and vehicle-injected animals (*F*_(1,128)_ = 10.63, *p* = 0.001). Interestingly, vagotomized animals with experimental inflammation prefer to explore more (*F*_(1,128)_ = 10.55, *p* = 0.001) and spent more time (*F*_(1,128)_ = 8.18, *p* = 0.005) in ending of the opposite, right closed arm.

Preferences for right or left closed arm are not associated with the animal choice of first entry to the left or right closed arm (χ^2^_(3,48)_ = 4.57, *p* = 0.206).

## Discussion

It is commonly accepted that the body, in situations when it is responding to threat, activates compensatory mechanisms. The aim of our study was to examine whether such mechanisms occur during infection to compensate for vagotomy-derived dysfunction of immune to CNS communication.

### Lack of vagal immune function is compensated by an increase of peripheral PGE2 concentration

According to mechanisms suggested by Tracey (2009), a high plasma IL-6 level is associated with low activity of vagus nerve [54]. In our study, we observed an insignificant tendency (*p* = 0.07) for slightly increased basal level of IL-6 in vagotomized and vehicle-injected rats. According to Tracey, exacerbation of mechanisms of innate immunity due to lack of tonic, immunosuppressive vagal activity could lead to elevated IL-6 plasma level. This suggestion supports the results shown by Calleja-Castillo et al. (2013) who demonstrated that unilateral cervical vagotomy of the right branch of the vagus led to an increase of plasma IL-6 level [55]. Alterations of IL-6 level after intraperitoneal injections of LPS in the present study are also consistent with those observed by Hansen et al. (2000) [56]. Comparing time after injection and the dose of LPS (in case of Hansen’s experiment it was 100 μg/kg), we also observed that vagotomy did not prevent the peripheral changes in IL-6 level in experimental inflammatory responses; however, we have shown that it generally significantly attenuated them. This observation is particularly important because IL-6 plays a role in HPA activation and fever induce [57]—according to this information, we should expect that HPA axis activity (indirectly measured as an corticosterone concentration changes) will be lowered in vagotomized group. Such results was presented in Borovikova et al. (2000) after cervical vagotomy [58]; however, in our study after less invasive, full recovered subdiaphragmatic vagotomy, corticosterone concentration after LPS injection was at the same level in both, vagotomized and sham animals. Such result suggests that HPA axis activity was at the same level despite the lack of vagal immunosensory signal and despite the lowered IL-6 concentration. It is also worth to note that IL-6 is a proinflammatory cytokine, produced quickly and transiently in response to infections and tissue damage. It stimulates acute phase reactions, hematopoiesis, and immunological reactions [59]. It also activates the B lymphocytes, leading to their differentiation and antibodies synthesis. Additionally, IL-6 positively affects the survival of plasmocytes which also have the ability to produce antibodies [60]. These facts indicate that IL-6 stimulate synthesis of the antibodies that are responsible for the course of the humoral response of the immune system. Decreased IL-6 plasma concentration, during inflammatory response, may indicate for intensification of non-specific processes in the early phases of the inflammation.

Our hypothesis assumes that appropriate level of neuro-immune communication, necessary for proper control of infection, was permitted by an increased level of plasma prostaglandins and they direct interaction with BBB vascular endothelial cells or omitting the barrier in circumventricular organs. Indeed, in the present study, we observed that plasma PGE2 concentration after LPS injection was increased in vagotomized compared to sham-operated rats. Our results are consistent with those presented by Matsumura et al. (2000) and their observation of increased PGE2 level in cerebrospinal fluid after LPS injection, 5 h after vagotomy [61]. Such a short time after surgery could suggest that this is a result of intraoperative tissue damage. However, we observed a similar result in blood plasma 4 weeks after operation. This implicates the participation of cyclooxygenase-dependent pathway in compensation for the lack of vagal immunosensory and immunosuppressive functions.

There is two possible explanation of the increased PGE2 concentration. First one applies anti-inflammatory function of the vagus nerve. It is involved in two, immunosuppressive mechanisms: (1) activation of the HPA axis and subsequent peripheral glucocorticoids release and (2) the reflexive, vagal anti-inflammatory cholinergic pathway [62, 63]. In our experiment, vagotomy did not affect an increase of corticosterone in blood plasma after LPS administration. Accordingly, we can assume that the observed PGE2 plasma concentration changes were associated with decreased immunosuppressive activity of the cholinergic anti-inflammatory pathway [64, 65]. Second explanation can be attributed to the effect of vagotomy on immune cells populations and mentioned promoting of non-specific, cellular immune response during infection. It is known that the lack of vagal signaling increases the proliferation of CD4^+^ lymphocyte precursors [42]. This fact and high PGE2 levels (probably increased as a result of lack of immunosuppressive, cholinergic anti-inflammatory pathway activity) influences T lymphocytes differentiation into Th2 lymphocytes [65], indicating that vagotomy preferentially promotes secreted immune signals as compared to cellular responses. PGE2-stimulated Th2 lymphocytes may in turn release large amounts of anti-inflammatory cytokines such as IL-4 that can stimulate cytotoxicity and phagocytosis of monocytes and macrophages, simultaneously inhibiting the release of proinflammatory cytokines from those cells [66, 67]. PGE2 itself may also modulate the range of cytokines released by macrophages (which can also release PGE2) [68]. This partially auto-regulative network may be a potential mechanism which is compensating for the lack of vagal immunosuppressive function at the local level. At the neuro-immune communication level, high PGE2 concentrations can reach the CNS and transfer inflammatory information through transport proteins in membranes of endothelial cells of the BBB [69] and circumventricular organs [70]. As we have demonstrated, this signal is more intense in vagotomized animals, putatively compensating for the lack of immunosensory signals from the vagus nerve. This mechanism, in our opinion, ensures continuity of immune to neural communication during inflammation in vagotomized animals, which is additionally confirmed by unchanged HPA axis activity.

### Changes in central neurotransmission caused by subdiaphragmatic vagotomy are generally normalized during peripheral inflammation

In light of the growing interest in the use of vagal activity in the context of various diseases, it is particularly important to define its role in regulating brain neurotransmitters, where disruption may result in biochemical-hormonal and psycho-behavioral alterations. The vagus nerve may indeed affect the functioning of many brain structures due to its numerous neuroanatomical connections via *nucleus tractus solitarii* (NTS) [71–75].

In the current experiment, we analyzed concentrations and utilization indexes (metabolism marker) of main, monoaminergic neurotransmitter in limbic areas and the cortex. Changes of concentration of all tested monoamines were area-specific and we cannot indicate any noticeable trends. On the other hand, analysis of monoaminergic utilization indexes allows us to define such general tendencies. We did not observe significant trends in noradrenaline or dopamine relative to DOPAC utilization index level in examined brain areas. However, in vagotomized animals, we observed a general trend for increased dopamine relative to HVA utilization index, and this tendency was accompanied by a decrease in the serotonin utilization index. It is noteworthy that after LPS administration, vagotomy-induced disruptions of 5HIAA/5HT and HVA/DA ratios were neutralized, reaching similar values as in the analogous, sham-operated group. It is possible that this “normalization” of the indexes is associated with the aforementioned elevated plasma PGE2 in vagotomized animals after LPS injection. A potential explanation of this phenomenon is related to the influence of PGE2 on NTS activity. NTS is a brainstem area which contain numerous sensory nerve endings, including vagal, and in turn, NTS has rich ascending, direct, and indirect, projections reaching numerous structures related to various aspects of functioning in disease: emotions, locomotor activity, and food intake, to name a few. NTS is in fact also one of the main binding sites of PGE2 within the brainstem [76]. Plasma PGE2 can activate endothelial cells of the BBB, and stimulate the release of secondary mediators to the CNS, or through direct access to the CNS through the circumventricular organs. PGE2 may be also released into the CNS parenchyma as secondary mediators by activated macrophages and glial cells. Physiological effects of PGE2 depends on the EP receptor (EP1–4) it binds [77] and within the NTS, a high expression of neuroprotective EP4 [78] and EP3 [79] has been observed.

The association of PGE2 signal and dopaminergic systems in the brain with the activity of the vagus nerve is supported by existing literature. Surówka et al. (2015) reported that VNS significantly influenced the lipid composition within the brain structures associated with the dopaminergic system [80]. VNS also caused an increase of glutamate concentration within the NTS [81] and decreased activity of the dopaminergic system [82]. As reported by Marty et al. (2008), secretion of glutamate by presynaptic membrane of sensory vagal neurons in NTS region is inhibited by PGE2 binding to EP3 receptors. At the same time, PGE2 stimulates the secretion of glutamate from local NTS cells by influencing other types of EP receptors; however, the author does not specify which ones [83]. Marty’s observations confirm the reports of Sekiyama et al. (1995) that PGE2 facilitates stimulating glutamatergic transmission in the NTS by increasing the release of this neurotransmitter from synaptic vesicles [84]. A similar physiological effect occurs in the paraventricular nucleus of the hypothalamus (PVN), where PGE2, likely through the EP1 and EP3 receptors [85], attenuates inhibitory GABAergic transmission [86]. The results of our study complement and simultaneously confirm the mutual influence of vagus nerve activity and PGE2 on the brain neurotransmission, particularly dopaminergic, during inflammation.

### Subdiaphragmatic vagotomy modulate behavior in EPM test

Trends observed in monoaminergic brain activity involve mainly the amygdala and hypothalamus, brainstem and motor cortex, and to a lesser extent on the prefrontal medial cortex. The amygdala is mainly responsible for generating negative emotions, defensive reactions, and processing of social information [87, 88]. The hypothalamus is a first component of stress axis, which is also crucial in immune response modulation [89]. This area is also crucial for body homeostasis at many levels—it is a neuroendocrine regulator for pituitary gland. It takes a part in the control of hunger, thirst, sleep, and body temperature [90]. The motor cortex is associated with physical activity. The brainstem contains many areas necessary for maintaining vital functions but it is also a place of integration of sensory and motor activity. The prefrontal medial cortex takes a part, i.e., in planning of movement and it can attenuate intensive emotional states [91]. Neurotransmission changes within those structures suggest possible behavioral implications.

It turns out that subdiaphragmatic vagotomy did not prevent the LPS-induced decrease of locomotor activity or risk assessment. Such result is compatible with aforementioned “normalization” of vagotomy-changed neurotransmission and HPA axis activity. More interestingly, vagotomy itself caused the same behavioral effect. In other words “healthy” rats after vagotomy behaved in our test similar to rats with developing inflammation. Such results could suggest that there is no cross-effect of vagotomy and inflammation; however, data indicates a slight increase of anxiety level in first minute of the test in LPS-injected vagotomized animals.

Heat map visualization and analysis of non-standard parameters (time spending in and number of entries to endings of the closed arms) reveals very interesting implications of subdiaphragmatic vagotomy. At first, we would like to note that after each test, arena was cleaned to remove scent of the animal before another test. However, rat’s sense of smell is very keen and side preference within the closed arms could reflect social proclivities or anxieties influenced by inflammatory processes. Surprisingly, such an unexpected “complication” allowed us to note some social-behavior results, though we will acknowledge that these are speculative and should be interpreted with caution. In our test, sham-operated animals displayed no preference for the end of the right or left closed arm. However, slightly higher number of entries to right one could indicate increased interest to this specific area. This observation is consistent with Edwards’s (1988) result, that uninfected animals showed more social investigatory behaviors with simultaneously lower touching episodes to infected animals [92]. Consequently, slightly increased exploration of the end of left closed arm by the sham group is visible at the heat map. Both sham-LPS-injected and vagotomized-vehicle-injected animals also spend more time in the ending of the left closed arm. Surprisingly, vagotomized animals in the LPS condition spent more time in the opposite area. This result we would like to interpret according to Loehle (1995) which had summarized the literature about the social barriers as a one of the physical barriers for pathogen transmission. He points out that social avoidance during disease episode is bidirectional—including both avoiding by healthy conspecifics and voluntary isolation of a sick animal [93]. Knowledge that sickness signals include odor allow us to interpret our observations with social avoidance.

We observed animal behavior 120 min after injection of LPS—time needed for observations pronounced changes of inflammatory markers in plasma of sham animals—with preserved proper function of the anti-inflammatory vagal mechanisms. Vagotomized animals react with higher PGE2 level and we think that their social isolation was caused by decreased fettle—it is known that PGE2 is involved in behavioral changes during disease but most of all in thermoregulatory processes [68, 94, 95]—EP3 receptor is involved in fever inducing [96]. Observed physiological and resulting behavioral changes after vagotomy may have important role for preventing infection spreading in situation when animals have impaired ability to properly control inflammatory response, especially given Hart’s (1988) understanding of sickness behavior as an adaptive “highly organized behavioral strategy, that is at times critical to the survival” [22, 93].

## Conclusions

Response of vagotomized animal to pathogens defined as a cytokines release, activation of HPA axis, and specific level of CNS monoaminergic transmission was almost the same like in sham-operated rats. Subdiaphragmatic vagotomy in association with inflammation caused specific changes of dopaminergic and serotoninergic concentration and metabolism in select brain limbic areas. It is possible that it can also intensify some of the sickness behavior.

**Fig. 9:**
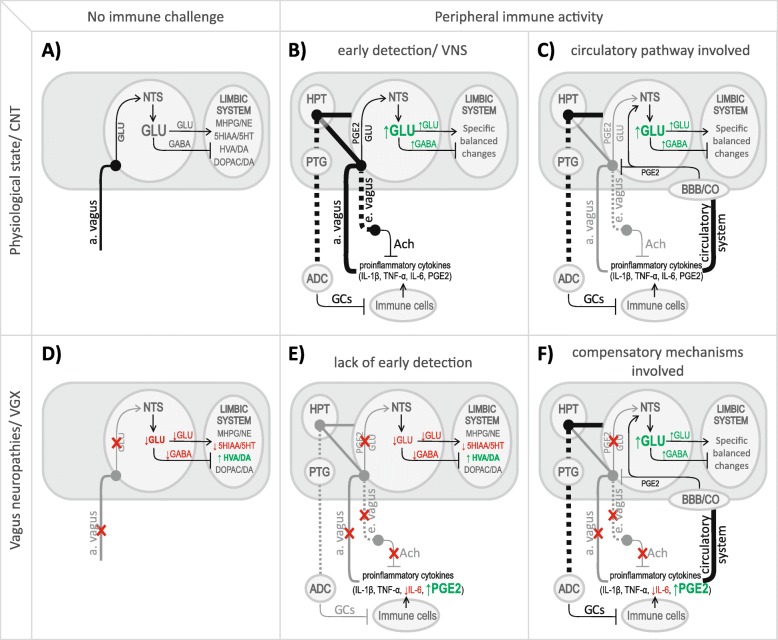
CNS (central nervous system), NTS (nucleus tractus solitarii), HPT (hypothalamus), PTG (pituitary gland), ADC (adrenal cortex), BBB (blood-brain barrier), CVO (circumventricular organs), GLU (glutamate), GABA (gamma-aminobutric acid), PGE2 (prostaglandin E2), Ach (acetylcholine), GCs (corticosteroids), IL-1β (interleukin 1β), IL-6 (interleukin 6), TNF-α (tumor necrosis factor α), upwards arrow (increase/green), downwards arrow (decrease/red), solid lines (afferent signaling), dotted lines (efferent signaling), gray lines (inactive pathways), black lines (active pathways). At physiological conditions (**a**), afferent vagal signaling provides an appropriate level of stimulating GLU within the NTS [81]. In the case of peripheral inflammation (**b**), the afferent of the vagus nerve is stimulated among others by IL-1β, that activating COX-2 and PGE2 synthesis in the presynaptic terminals of this fibers in within the NTS [5, 64]. PGE2 secreted by the vagus nerve stimulates other neurons in the NTS to secrete additional portions of GLU. This mechanism permits quick “detection” of peripheral inflammation and rapid “launching” of the HPA axis. This signal is also transmitted to the other CNS structures, resulting in specific changes in activity of neurotransmission systems, which, while remaining physiologically balanced, allows for a correct central reaction to the inflammation development. When inflammatory mediators reach the brain via the bloodstream (**c**), they stimulate vascular endothelial cells, tissue macrophages, and glial cells in the brain to release PGE2, which stimulates NTS cells to release GLU [84]. Glutamatergic information from the vagus nerve becomes unnecessary and could threaten to overstimulate (glutamate excitotoxicity, [97]), which is why it is limited by PGE2 acting on the EP3 receptors on the presynaptic membrane of the afferent endings of the vagus nerve [83]. It is possible that when afferent vagal signaling is absent (**d**), GLU concentration in the NTS may be chronically decreased. This could lead to disturbances in the functioning of major neurotransmitter systems. As demonstrated in our experiment, in some brain areas, serotonergic activity decreased. There was also a strong increase in extracellular dopamine metabolism likely secondary to a lack of glutamatergic stimulation of inhibitory GABAergic neurons. It is also possible that brain releases additional portions of the neurotransmitter (dopamine) into the synaptic cleft, to maintain the neural activity at an appropriate, physiological level, thus compensating for the insufficient level of GLU in NTS. Under such conditions, when infection appears in the periphery (**e**), limited sensory and immunosuppressive functions of the vagus nerve renders delayed detection of the infection. This may lead to the aforementioned significant increase in PGE2 in blood plasma. The inflammatory signal is transferred to the brain through the BBB and CVO (**f**) causing an increase in the GLU content in NTS, and then an apparent “normalization” of the neurotransmitter activity of the limbic system areas. The HPA axis is also activated. This model is supported by fact that PGE2 is necessary to cholinergic anti-inflammatory pathway functioning [98]

Despite vagotomy, communication between immune system and CNS is largely preserved via activation of alternative communication mechanisms. These mechanisms likely involve peripheral PGE2 synthesis, compensating for a lack of immunosensory vagal signal. We hypothesized that diminished immunosensory and immunosuppressive vagal activity in vagotomized animals prevent an appropriate, rapid detection and attenuation of inflammation in the peritoneal cavities (Fig. 9). It may lead to increased release of PGE2, which we observed. The most likely source of this peripheral PGE2 seems to be activated immune cells, in particular monocytes and macrophages; however, this issue is still unsolved. We do not know whether this increased PGE2 level is caused by increased activity or expression of PGE2-synthesizing enzymes in PGE2-releasing immune cells. Increased PGE2 level may be also caused by an increase in the number of these cells or, finally, it is simply an effect of long-term lack of vagal immunosuppression at early stages of inflammation.

## Abbreviations

5HIAA: 5-Hydroxyindoleacetic acid
5HT: Serotonin
ACH: Acetylcholine
ADC: Adrenal cortex
AM: Amygdala
ANOVA: Analysis of variance
BBB: Blood-brain barrier
BS: Brainstem
CAN: Central autonomic network
CNS: Central nervous system
COX: Cyclooxygenase
CTX: Motor cortex
CVO: Circumventricular organs
DA: Dopamine
DOPAC: 3,4-Dihydroxyphenylacetic acid
ELISA: Enzyme-linked immunosorbent assay
EPM: Elevated plus maze
GABA: Gamma-aminobutyric acid
GCs: Corticosteroids
GLU: Glutamate
HI: Hippocampus
HPA axis: Hypothalamic-pituitary-adrenal axis
HPLC-MS: High-performance liquid chromatography with mass spectrometry
HPT: Hypothalamus
HVA: Homovanillic acid
IL: Interleukin
LPS: *E*. *coli* lipopolysaccharide
MANOVA: Multivariate analysis of variance
MHPG: 3-Methoxy-4-hydroxyphenylglycol
NE: Noradrenaline
NTS: Nucleus tractus solitarii
PAG: Periaqueductal gray
PFM: Prefrontal medial cortex
PGE2: Prostaglandin E2
PTG: Pituitary gland
PVN: Paraventricular nucleus of the hypothalamus
RIA: Radioimmune assay
RP-HPLC-ED: Reversed-phase, high-performance liquid chromatography with electrochemical detection
TNF-α: Tumor necrosis factor α
VGX: Vagotomy
VNS: Vagus nerve stimulation
